# Is 25OH Vitamin D Excess before 36 Weeks Corrected Age an Independent Risk Factor for Bronchopulmonary Dysplasia or Death?

**DOI:** 10.3390/nu15204423

**Published:** 2023-10-18

**Authors:** Sophie Laborie, Maxime Bonjour, Justine Bacchetta, Mathilde Mauras, Marine Butin

**Affiliations:** 1 Service de Réanimation Néonatale et Néonatologie, Hôpital Femme Mère Enfant, Hospices Civils de Lyon, 69677 Bron, France; marine.butin@chu-lyon.fr; 2 Service de Biostatistique-Bioinformatique, Pôle Santé Publique, Hospices Civils de Lyon, 69003 Lyon, France; maxime.bonjour@chu-lyon.fr; 3 Faculté de Médecine Lyon Est, Université Claude Bernard Lyon 1, 69373 Lyon, France; justine.bacchetta@chu-lyon.fr; 4 Service de Néphrologie, Rhumatologie et Dermatologie Pédiatriques, Centre de Référence des Maladies Rares du Calcium et du Phosphore, Hôpital Femme Mère Enfant, Hospices Civils de Lyon, 69677 Bron, France; 5 INSERM 1033, Prévention des Maladies Osseuses, 69372 Lyon, France; 6 Service Pédiatrie B, Hôpital Nord, CHU de Saint-Etienne, 42270 Saint Priest en Jarez, France; mathilde.mauras@chu-st-etienne.fr; 7 Centre International de Recherche en Infectiologie, INSERM U1111, CNRS UMR5308, Ecole Normale Supérieure de Lyon, 69365 Lyon, France

**Keywords:** vitamin D, premature infants, bronchopulmonary dysplasia, low-birthweight infant, very-low-birthweight infant

## Abstract

Low 25-Hydroxyvitamin D (25(OH)D) in preterm infants is a risk factor for bronchopulmonary dysplasia (BPD), but increased supplementation failed to demonstrate a beneficial effect on BPD. In neonatal animal models, deficiency and excessive vitamin D exposure have been associated with increased mortality and histological alterations in the lung evocative of BPD. Our hypothesis is that 25(OH)D levels ≥ 120 nmol/L are also a risk factor for BPD or death. This retrospective single-center cohort study included only infants born at <31 weeks gestational age without major malformations with at least a determination of 25(OH)D at <36 weeks corrected age and no determination <50 nmol/L. Routine 25(OH)D determination was performed at 1 month and monthly thereafter. A total of 175 infants were included. Infants with BPD or who died had a significantly lower term and weight, but a similar frequency of 25(OH)D ≥120 nmol/L (50.5% vs. 43.9%, *p* = 0.53). The logistic regression identified weight (OR 0.997, 95% CI [0.995–0.998]) and term (OR 0.737, 95% CI [0.551–0.975]) as significantly associated with BPD or death; the occurrence of excessive 25(OH)D was not significantly associated (OR 1.029, 95% CI [0.503–2.093]). The present study did not demonstrate any significant association between excessive 25(OH)D after one month of age and BPD or death.

## 1. Introduction

Bronchopulmonary dysplasia (BPD) is a frequent and sometimes severe complication of premature infants with long-term consequences [1]. Vitamin D is implicated in lung development, as demonstrated by multiple animal studies in rodents [2], and supplementation with low doses of native vitamin D in rodent pups exposed to hyperoxia is reported to attenuate the histological and some biochemical markers of BPD [3]. Low concentrations of 25-Hydroxyvitamin D (25(OH)D) at birth and at one month of age have been associated with increased risk of BPD as demonstrated by a meta-analysis [4] and a study adjusting for the factors known to be associated with BPD [5]. Furthermore, low 25(OH)D at one month of age has also been associated with increased risk of bronchopulmonary dysplasia [6]. However, studies investigating high-dose supplementation (compared with low-dose) failed to demonstrate any significant effect on the frequency of BPD [7,8] but have found high 25(OH)D concentrations in the groups exposed to high doses [8,9,10]. The data on the consequence of vitamin D excess in this population (except for the risk of nephrocalcinosis and/or hypercalcemia) are sparse [11]. In rodents receiving vitamin D in excess during gestation and lactation, pups had an abnormal lung histology; there was a greater mean linear intercept, a greater total respiratory system resistance, and a lower basal proliferation of their lung mesenchymal stem cells with a lower adipogenic and a greater myogenic potential [12,13]. Furthermore, in a model of bronchopulmonary dysplasia, neonatal pups exposed to oxygen receiving high doses of 1,25-di-Hydroxyvitamin D (1,25(OH)_2_D) from the first day of life exhibited higher mortality and an altered lung histology (increased mean linear intercept, a decreased angiogenesis, and increased proinflammatory factors) when compared with animals receiving low doses [14]. Recent studies have demonstrated a high frequency of excessive levels of 25(OH)D in preterm infants with supplementation recommended at that time [15,16,17]. Our hypothesis was that these excessive 25(OH)D levels in very and extremely preterm infants may be deleterious to pulmonary development and may therefore be implicated in the pathogenesis of BPD. The primary objective of this study was, therefore, to determine whether excessive 25(OH)D levels are an independent risk factor for BPD or death.

## 2. Materials and Methods

In this retrospective cohort study, all infants born at <31 weeks gestational age between January 2018 and December 2019 were eligible for inclusion if they were hospitalized before 3 days of life and for at least 10 days in the neonatal intensive care unit (NICU) in the Hospital Femme Mere Enfant, Bron, France, and presented no major congenital malformation. They were included if they had at least a 25(OH) D determination at <36 weeks corrected age. They were excluded if they presented at least a 25(OH)D determination <50 nmol/L.

**Figure 1:**
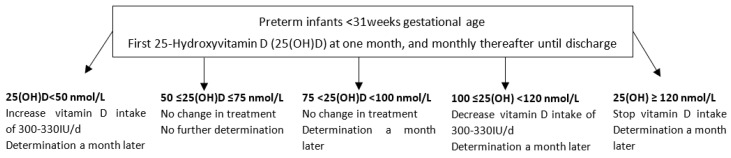
Local protocol of adaptation of vitamins in extremely and very preterm infants.

In this NICU, preterm infants receiving parenteral nutrition were supplemented with Cernevit (Baxter, Guyancourt, France) in an amount of ¼ vial daily (containing 55 IU cholecalciferol). When parenteral nutrition was stopped, infants with a weight below 1 kg received Sterogyl (DB pharma, La Varenne-St-Hilaire, France) in an amount of 3 drops daily (1200 IU ergocalciferol) while infants above 1 kg received Uvesterol ADEC (Crinex, Montrouge, France) in an amount of 0.3 mL daily (containing 1000 IU ergocalciferol). These supplementations were following or slightly above the European Society of Paediatric Gastroenterology, Hepatology and Nutrition’s recommendations at that time of 800–1000 IU daily during enteral nutrition and more than 30 IU during parenteral nutrition [18,19]. A routine determination of 25(OH)D was recommended in our unit at one month of age and monthly thereafter until discharge with a protocol for adaptation of the dose (Figure 1). The objective was to maintain 25(OH)D ≥ 50 and < 120 nmol/L.

The main outcome was BPD or death at 36 weeks corrected age. BPD was defined as the need for supplemental oxygen or respiratory support to maintain a saturation equal to or above 90% at 36 weeks corrected age with radiological evidence of parenchymal lung disease [20]. In the description of the population, it was classified into three grades according to Jobe et al. [21].

The main early predictive factors of BPD reported in recent studies were collected [22,23,24,25]: multiple gestations, antenatal corticosteroids, spontaneous delivery, gestational age at birth, birthweight, Apgar at 5 min (in categories 8 to 10, 4 to 7, 0 to 3), sex, respiratory support during the first 24 h (classified in 3 groups—mild FiO2 < 30% and noninvasive ventilation, moderate FiO2 < 30% and mechanical ventilation, severe FiO2 ≥ 30% and mechanical ventilation—as proposed by Baud et al. [22]), and breastfeeding defined as receiving any mother’s milk. Ethnic origin was not available; however, Baud et al. excluded it from their final predictive model in a French population [22].

“Small for gestational age” was defined as a weight below the tenth percentile according to the Fenton curves [26]. Enterocolitis was considered present if a grade of 2 or above was observed.

Data were extracted from electronic medical charts (IntelliSpace Critical Care and Anesthesia prescription software, Philips, Suresne, France) and completed with the discharge letter when infants were transferred to another hospital or another unit.

The number of subjects was calculated based on the unpublished results of a pilot study [27]. Based on the results of the multiple logistic regression simulation taking into account confounding parameters (term, spontaneous birth, and sex) and excessive 25(OH)D concentration, the number of infants necessary to find an OR of 2.8 for BPD and a 25(OH)D concentration association was 176, with a power of 80% and an alpha risk of 0.05.

The quantitative variables were described using the mean and standard deviation (SD), and qualitative variables using the number of patients and frequency (%) of each modality.

Patients were stratified according to the maximal 25(OH)D concentration between 1 month of life and 36 weeks corrected age (excessive [any determination ≥ 120 nmol/L] [28] or normal [all determinations ≥50 to <120 nmol/L]). Patients with BPD or who died at 36 weeks corrected age were compared with other patients using the Wilcoxon or chi-squared tests, as appropriate. The analysis of the association between BPD and excessive 25(OH)D concentration was investigated using a logistic regression model constructed using backward stepwise selection.

25(OH)D concentration was measured using a chemiluminescent microparticle immunoassay with an Isys analyzer (Immunodiagnostic Systems, Pouilly-en-Auxois, France).

This study was approved by the institutional review board (*Comité Scientifique et Éthique*) of the Hospices Civils de Lyon on 18 January 2023 (number 23_076). It also received the approval of the national data protection commission (Commission Nationale de l’Informatique et des Libertés; number 23_5076). According to French law, parental informed consent was not necessary, but all parents were informed and could refuse the participation of their infant.

This study is registered in ClinicalTrials.gov/study/NCT05944055 (accessed on 17 July 2023).

## 3. Results

### 3.1. Population

#### 3.1.1. Study Flow-Chart

**Figure 2:**
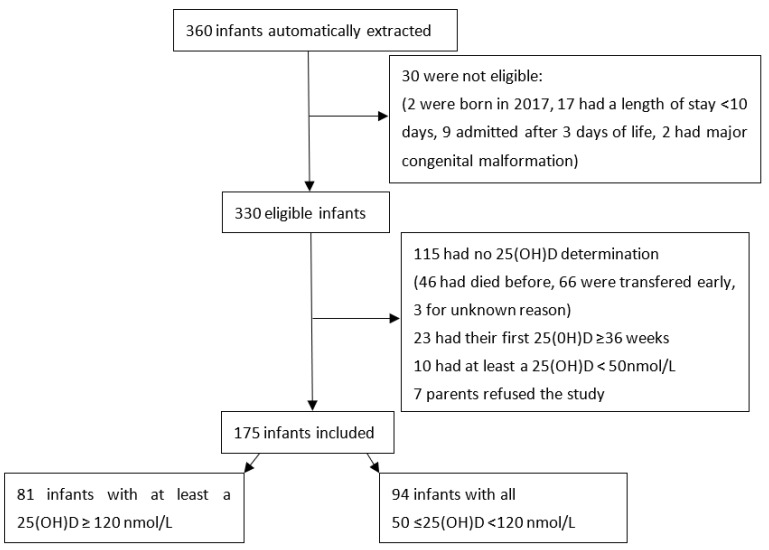
Study flow-chart.

The study flow-chart is presented in Figure 2.

#### 3.1.2. Description of the Population

A total of 175 infants were included, of which 81 (46.3%) had at least one 25(OH)D ≥ 120 nmol/L, and for the remaining 94 (53.7%) 25(OH)D was always between 50 and 120 nmol/L. The obstetrical characteristics of the included population and according to 25(OH)D concentration are described in Table 1.

The main neonatal characteristics of the included population and according to 25(OH)D concentration are presented in Table 2.

### 3.2. Outcomes

The main outcomes of the cohort according to 25(OH)D concentration are presented in Table 3.

### 3.3. Analysis

Univariate analysis found that term (BPD or death: median 26.50, interquartile range (25.57–27.79) vs. no BPD or death: median 28.29 interquartile range (27.36–29.43), *p* < 0.001) and weight (BPD or death: median 775 g, interquartile range (635–892) vs. no BPD or death: median 1050 g, interquartile range (900–1232), *p* < 0.001) were significantly different between infants with BPD or death and those without. The occurrence of 25(OH)D ≥ 120 nmol/l (50.0% vs. 43.9%, *p* = 0.53) was not significantly different between the two groups (with BPD or death and without). Multiple pregnancy, Apgar score, sex, any mother’s milk given, spontaneous birth, and maximum ventilation during the first 24 h of life were not significantly different between groups.

The results of the multivariable analysis are presented in Table 4 with the full saturated model and the final model. In the final model, term (OR 0.737, 95% CI [0.551–0.975], *p* = 0.035) and weight (OR 0.997, 95% CI [0.995–0.998], *p* = 0.001) were significantly associated with BPD or death; there was no significant association with any 25(OH)D determination ≥120 nmol/L (OR 1.029, 95% CI [0.503–2.093], *p* = 0.936).

A post hoc analysis was performed to evaluate whether the occurrence of a 25(OH)D above 150 nmol/L was associated with the occurrence of BPD or death. Again, only term (OR 0.736, 95% CI [0.551–0.971], *p* = 0.033) and weight (OR 0.997, 95% CI [0.995–0.999], *p* = 0.001) were significantly associated; there was no significant effect of 25(OH)D >150 nmol/L (OR 1.291, 95% CI [0.558–2.982], *p* = 0.548).

## 4. Discussion

As reported herein, a high frequency of excessive 25(OH)D levels with high enteral intakes of vitamin D has been reported [15,16,17] and the recommendations from the European Society of Paediatric Gastroenterology, Hepatology and Nutrition have been updated with a decreased recommended intake during enteral nutrition (400–700 IU daily) and an increased recommended intake during parenteral nutrition [29,30].

Unlike the results obtained in an animal model [12,13,14], the present study did not find that excessive 25(OH)D concentration was a risk factor for BPD or death. This result may be related to the temporality of the excessive concentration, as in animal studies native vitamin D was administrated throughout the gestation period [12,13] or 1,25(OH)_2_D was administrated immediately at birth [14]. With such early administration, the lungs are more immature and their development may be severely impaired. We chose to study 25(OH)D at the first month because the frequency of excessive concentrations at birth in preterm infants is very low [31,32,33]. In France, Courbebaisse et al. reported that in the general population of newborns, 93% of cord blood concentrations were below 75 nmol/L [34], and Papalia et al. reported that in infants born below 29 weeks gestational age, 74% had a cord blood concentration ≤75 nmol/L [35]. In France, the current recommendation for vitamin D supplementation during pregnancy is to administer 100,000 IU once during the seventh month of pregnancy. This recommendation was followed in 88% of the pregnant patients in a recent large cohort study [34] and may explain these results. Furthermore, the vitamin D intake during parenteral nutrition was low herein (55 IU daily), and the median duration of parenteral nutrition was 14 days. Taking into account these aspects and the results of the study reported by Fort et al. (who described the increase in 25(OH)D in preterm infants receiving 200, 400, and 1000 IU daily) [9], we estimated that the risk of early excessive concentration was low in the study population.

The upper limit of normal 25(OH)D was established in accordance with the current recommendation of the European Society for Paediatric Nephrology for infants with chronic kidney disease [28,36] and recent French and European recommendations for preterm infants [29,37]. It was justified by an increase in mortality with higher concentrations in the general population [38,39]. This threshold is reinforced by the results of a case series study that identified 16 preterm infants referred to nephrology clinics for symptomatic hypervitaminosis D with 25(OH)D concentrations between 119 and 350 nmol/L [11]. In two previous studies, higher concentrations were associated with a high frequency of hypercalciuria [15,16]. However, it remains possible that the effect of excessive 25(OH)D concentration on lung development necessitates concentrations above 120 nmol/L, although the results of the post hoc analysis with concentrations above 150 nmol/L do not support this hypothesis.

The next factor that could explain the discrepancy between the results observed in the animal model reported by Chen et al. [14] and the present study is that in this animal study the active form of vitamin D, namely 1,25(OH)_2_D, was used, whereas native vitamin D was used herein, according to clinical practice. Using 1,25(OH)_2_D, the physiological regulation of the production of 1,25(OH)_2_D is circumvented, even though the limiting factor of this synthesis as classically described is the availability of 25(OH)D in preterm infants [40], but the regulation in extremely and very preterm infants and in particular the function of the C3 epimers are still not fully elucidated [41,42]. The results of the studies reported by Wang et al. and Mandell et al. in rodent pups exposed to hyperoxia receiving native vitamin D demonstrated improved lung histology but 25(OH)D was measured at low and normal levels, not allowing conclusions on supraphysiological doses [3,43]. However, the results reported by Yurt et al. and Sakurai et al. using supraphysiological doses of native vitamin D during rat gestation demonstrated deleterious consequences on the lung with high doses even without oxygen exposure [12,13].

The main limitation of this study is the absence of determination of 25(OH)D at birth; some infants from both groups may have experienced an early deficiency in 25(OH)D, which is a recognized risk factor for BPD [4,6], and they may not be equally distributed between groups, decreasing the difference between groups for the primary outcome. Despite this limitation, these results are important because they show that in the absence of an early determination of 25(OHD), a 25(OH)D concentration above 120 nmol/L before 36 weeks corrected age is not a significant risk factor for BPD or death. In addition, there does not seem to be a great difference in terms of morbidity according to 25(OH)D concentration herein, although this was not formally tested to avoid multiplicity of comparisons. Another limitation is the retrospective nature of this study. Some variables such as ethnicity which are known to interfere with vitamin D metabolism [8] and BPD frequency [44] were not available, and the risk of bias was increased.

Further studies are necessary to determine the appropriate modalities of administration of native vitamin D in extremely and very preterm infants as it is a modifiable factor that could impact the risk of BPD [4,6] and the risk of sepsis [45,46,47,48], two essential factors for the future of premature infants, but also nephrological and bone-related outcomes. The prevention of vitamin D deficiency at birth and the effectiveness of treatment with native vitamin D in infants with depleted and normal levels at birth should be evaluated with a careful monitoring of respiratory and infectious outcomes. Retrospective studies may give us clues (indication and dosage regimen) for further randomized control trials.

## Figures and Tables

**Table 1:** Main obstetrical characteristics of the studied population.

Pregnancy Characteristics *		Excessive 25(OH)D N = 81	Normal 25(OH)D N = 94	Total N = 175
Parity	1	29 (35.8%)	40 (42.6%)	69 (39.4%)
	2	25 (30.9%)	31 (33.0%)	56 (32.0%)
	3	19 (23.5%)	14 (14.9%)	33 (18.9%)
	≥4	8 (9.9%)	8 (8.5%)	16 (9.1%)
	Unknown	0 (0.0%)	1 (1.1%)	1 (0.6%)
Multiple pregnancy		30 (37.5%)	25 (26.6%)	56 (32.0%)
Any hypertension during pregnancy		19 (23.5%)	21 (22.3%)	40 (22.9%)
Preterm premature rupture of membranes		26 (32.1%)	27 (28.7%)	53 (30.3%)
Any diabetes during pregnancy		8 (9.9%)	13 (13.8%)	21 (12.0%)
Histological chorioamnionitis		21 (25.9%)	13 (13.8%)	34 (19.4%)
	Unavailable	0 (0%)	3 (3.2%)	3 (1.7%)
Clinical chorioamnionitis		11 (13.6%)	7 (7.4%)	18 (10.3%)
	Unknown	1/1.2%)	3 (3.2%)	4 (2.3%)
Any antenatal corticosteroids		76 (93.8%)	83 (88.3%)	159 (90.9%)

* Data were available for all infants except if specified.

**Table 2:** Main neonatal characteristics of the studied population.

Neonatal Characteristics *	N (%), Except if Specified		Excessive 25(OH)D N = 81	Normal 25(OH)D N = 94	Total N = 175
Birth season	Summer		19 (23.5%)	32 (34.0%)	51 (29.1%)
	Fall		20 (24.7%)	26 (27.7%)	46 (26.3%)
	Winter		21 (25.9%)	18 (19.1%)	39 (22.3%)
	Spring		21 (25.9%)	18 (19.1%)	39 (22.3%)
Sex	Male		39 (48.1%)	51 (54.3%)	90 (51.4%)
Term (weeks)	Mean (SD)		27.58 (1.84)	27.88 (1.56)	27.74 (1.70)
Weight (g)	Mean (SD)		938 (272)	998 (305)	970 (291)
Height (cm)	Mean (SD)		35.05 (3.11)	35.15 (3.98)	35.11 (3.60)
Head circumference (cm)	Mean (SD)		24.83 (2.19)	25.44 (2.72)	25.16 (2,50)
Small for gestational age **			14 (17.3%)	17 (18.1%)	31 (17.7%)
Apgar at 5 min	8–10		55 (67.9%)	52 (55.3%)	107 (61.1%)
	4–7		21 (25.9%)	37 (39.4%)	58 (33.1%)
	0–3		4 (4.9%)	4 (4.3%)	8 (4.6%)
	Not available		1 (1.2%)	1 (1.1%)	2 (1.1%)
Maximum ventilation during the first 24 h	FiO2 < 30% and noninvasive ventilation		7 (8.6%)	6 (6.4%)	13 (7.4%)
	Assisted ventilation and FiO2 < 30%		0 (0.0%)	1 (1.1%)	1 (0.6%)
	Assisted ventilation or FiO2≥ 30%		74 (91.4%)	87 (92.6%)	161 (92.0%)
Parenteral nutrition (days)	Median		14	13	14
	Interquartile range		7–23	7–20	7–21
Enteral feeding	Maternal or donor milk		17 (21.0%)	19 (20.2%)	36 (20.6%)
	Mixed		50 (61.7%)	49 (52.1%)	99 (56.6%)
	Formula or donor milk		14 (17.3%)	26 (27.7%)	40 (22.9%)
Any mother’s milk given			66 (81.5%)	68 (72.3%)	134 (76.6%)
First determination of 25(OH)D (nmol/L)		Mean (SD)	139.4 (43.1)	86.1 (19.6)	110.8 (42.1)
Corrected age at first 25(OH)D determination		Mean (SD)	32.4 (1.9)	32.5 (1.5)	32.4 (1.7)
Second determination of 25(OH)D (nmol/L)			25 (30.9%)	20 (21.3%)	45 (25.7%)
		Mean (SD)	144.40 (29.58)	85.20 (17.76)	118.1 (38.7)
Corrected age at second determination of 25(OH)D		Mean (SD)	34.2 (1.2)	34.2 (1.5)	34.2 (1.3)

* Data were available for all infants except if specified. ** Weight below the tenth percentile according to Fenton curves [26]. SD: standard deviation.

**Table 3:** Main outcomes of the studied population.

Outcomes *	N (%), Except if Specified	Excessive 25(OH)D N = 81	Normal 25(OH)D N = 94	Total N = 175
Intraventricular hemorrhage	1	6 (7.4%)	11 (11.7%)	17 (9.7%)
	2	2 (2.5%)	4 (4.3%)	6 (3.4%)
	3	1 (1.2%)	1 (1.1%) 4 (4.3%)	2 (1.1%)
	4	3 (3.7%)	4 (4.3%)	7 (4.0%)
Cystic periventricular leukomalacia		0 (0.0%)	5 (5.3%)	5 (2.9%)
Retinopathy of prematurity	1	9 (11.1%)	15 (16.0%)	24 (13.7%)
	2	18 (22.2%)	14 (14.9%)	32 (18.3%)
	3	7 (8.6%)	5 (5.3%)	12 (6.9%)
	Unknown	9 (11.1%)	13 (13.8%)	22 (12.6%)
PDA requiring treatment		22 (27.2%)	30 (31.9%)	52 (29.7%)
Enterocolitis grade ≥2	2	0 (0.0%)	1 (1.1%)	1 (0.6%)
	3	0 (0.0%)	2 (2.1%)	2 (1.1%)
Number of sepsis events	1	23 (28.4%)	19 (20.2%)	42 (24.0%)
	2	4 (4.9%)	5 (5.3%)	9 (5.1%)
	3	1 (1.2%)	1 (1.1%)	2 (1.1%)
BPD grade	No BPD	18 (22.8%)	22 (23.9%)	40 (23.4%)
	Mild	29 (36.7%)	38 (41.3%)	67 (39.2%)
	Moderate	24 (30.4%)	31 (33.7%)	55 (32.2%)
	Severe	8 (10.1%)	1 (1.1%)	9 (5.3%)
Moderate or severe BPD or death		34 (42.0%)	34 (36.2%)	68 (38.9%)
Moderate or severe BPD		32 (39.5%)	32 (34.0%)	64 (36.6%)
Death before 36 weeks corrected age		2 (2.5%)	2 (2.1%)	4 (2.3%)

* Data were available for all infants except if specified. BPD: bronchopulmonary dysplasia; PDA: persistent ductus arteriosus.

**Table 4:** Multivariable analysis to evaluate the risk of bronchopulmonary dysplasia or death taking into account the classically described factors and the occurrence of a 25(OH)D ≥120 nmol/L.

Variables		OR	95%CI *	*p*-Value
Full model				
Any 25(OH)D determination ≥ 120 nmol/L		1.011	0.475–2.145	0.977
Weight (g)		0.997	0.994–0.999	0.019
Term (weeks)		0.661	0.436–0.989	0.046
Multiple pregnancy		1.881	0.855–4.248	0.120
Sex, female		0.530	0.242–1.134	0.106
Maximal ventilation during the first 24 h		0.838	0.210–3.743	0.806
Any mother’s milk administrated		1.237	0.520–3.011	0.634
Apgar at 5 min	8–10	1.000		
	4–7	0.912	0.402–2.041	0.823
	0–3	0.795	0.147–4.108	0.782
Spontaneous birth		0.631	0.223–1.770	0.381
Final model				
Any 25(OH)D determination ≥ 120 nmol/L		1.029	0.503–2.093	0.936
Weight (g)		0.997	0.995–0.998	0.001
Term (weeks)		0.737	0.551–0.975	0.035

* 95% CI: 95% confidence interval.

## Data Availability

The data presented in this study are available on request from the corresponding author.
